# Anesthetic Challenges and Management of Obstetric Emergencies in a Secondary Care Hospital in a Remote Mining Area: A Case Series

**DOI:** 10.7759/cureus.68675

**Published:** 2024-09-04

**Authors:** Joyanta Das Bhowmik, Dhirendra Kumar

**Affiliations:** 1 Anesthesiology, Tata Main Hospital, Noamundi, IND; 2 Surgery, Tata Main Hospital, Noamundi, IND

**Keywords:** cesarean section, anesthesia, fetal distress, resuscitation, hemodynamic, emergency

## Abstract

Obstetrical emergencies are life-threatening situations that can arise during pregnancy, labor, and post-delivery. Despite advances in the healthcare system, pregnancy-related complications like antepartum and postpartum hemorrhage, pre-eclampsia, and eclampsia are still the leading causes of maternal and fetal mortality in India. This case series describes anesthetic management in five obstetric cases for cesarean section and laparotomy and also highlights the magnitude of maternal and perinatal conditions following an event, with which they report to the hospital, where, in most of the cases, there was no time for optimization, and the lives of both the mother and fetus were at risk. It was a challenging task to deliver anesthesia services to these high-risk patients with complications and comorbidities, including but not limited to pregnancy-induced hypertension, severe anemia, and hemorrhage. A diligent approach, close collaboration with interdisciplinary teams, and intervention at the appropriate time ensured the best possible outcome.

## Introduction

Obstetric emergencies are life-threatening situations that develop during pregnancy, active labor, or after delivery [1]. Anesthesia for cesarean section (C-section) during obstetrical emergencies has very high mortality and morbidity rates for both mother and baby. Pregnant women are at increased risk of complications because of the physiological changes they experience during pregnancy, which result in a decreased threshold to hypoxia, a predisposition to hemorrhage, and a difficult airway due to edema [2]. According to the National Family Health Survey 5, the rate of C-section deliveries has been increasing in India, accounting for 17.2% in 2015-2016 and 21.5% in 2019-2021 [3]. C-section deliveries are mostly categorized as either emergency or elective surgery, irrespective of the time available for delivery of the baby. However, presently, as per the guidelines set by the National Institute for Health Care Excellence, the urgency of cesarean birth is further categorized as follows: emergency, with an immediate threat to the life of the woman or fetus (e.g., suspected uterine rupture, major placental abruption, cord prolapse, fetal hypoxia, or persistent fetal bradycardia); urgent, with some compromise of the mother or fetus that is not immediately life-threatening; scheduled, with no maternal or fetal compromise but early birth required; and elective, with the birth timed to suit the mother or healthcare provider [4]. The outcome of the mother and baby also depends on the timeline between a decision being made and the delivery of the baby, which is known as the decision delivery interval (DDI) [5]. For emergency and urgent cesarean birth, a C-section is to be conducted as soon as possible in most cases, namely, within 30 minutes and 75 minutes of making the decision, respectively. Factors that can affect DDI include the availability of staff resources, hospital infrastructure, and other supporting groups (e.g., pathology and blood bank services). Therefore, the choice of anesthesia technique depends on the indication of the C-section and DDI [5]. Interventions for enhanced recovery after surgery protocol like prophylactic antibiotics, preloading with intravenous fluid, active patient warming, neuraxial block, skin contact/breastfeeding, multimodal analgesia, and early mobilization in these patients posted for C-section may further augment recovery.

## Case presentation

This case series is a collection of obstetric emergencies, each with unique challenges and management strategies. We aim to share our experiences and insights from managing complex situations in a remote area, highlight the importance of a multidisciplinary team approach, and discuss evidence-based management approaches and their applications.

Case 1

A 28-year-old woman from a nearby village, second gravida, at 36 weeks of pregnancy presented to our emergency department on April 6, 2024, with a history of acute onset abdominal pain and vaginal bleeding. Prior to this visit, she did not attend any antenatal checkup (ANC). Her general condition was poor, with a body weight of 42 kg and a short stature. On examination, she had severe pallor, tachycardia with a pulse rate of 118/min, and a low blood pressure of 72/46 mmHg. Airway and other systemic examinations were normal. Abdominal examination revealed a boggy uterine feeling with audible fetal heart sounds (FHSs). She was diagnosed with uterine rupture and immediately posted for an emergency C-section. Three units of whole blood were ordered on an urgent basis, and a minimum blood investigation order comprising complete blood count, renal function test (RFT), liver function test (LFT), grouping and crossing matching, bleeding time, and clotting time was sent. The patient was given fluid resuscitation with 250 mL of colloid along with injections of pantoprazole, metoclopramide, and ondansetron for aspiration prophylaxis. The blood investigation revealed an exceptionally low Hb% (4.5 gm/dL), a slightly high total leucocytic count, and a normal total platelet. The patient’s kidney function and liver function parameters were normal, as were her total platelet count, bleeding time, and clotting time. General anesthesia with rapid sequence intubation was planned, and preoxygenation was done for three minutes with a tight-fitting mask. Induction was conducted with a titrated dose of ketamine (50 mg) and succinylcholine (50 mg). Cricoid pressure was applied and continued until intubation was confirmed by ETCO2 measurement. Maintenance of anesthesia was continued with vecuronium, along with nitrous oxide and oxygen (1:1), without the use of any inhalational agent. Paracetamol infusion 1 gm and tramadol 50 mg (after the delivery of the baby) were used as analgesics. Intraoperative findings were suggestive of an impending rupture with a large retroplacental hematoma. A moderately depressed live male baby with an Apgar score of 6 was delivered. The baby was resuscitated and kept under close observation. Two units of whole blood were transfused, followed by a titrated dose of dopamine, and a dobutamine infusion was given during the intraoperative period. A combination of both dopamine and dobutamine was administered after fluid resuscitation and volume replacement for short-term clinical recovery facilitated by the visible effect on hemodynamics by enhancement of cardiac output. After the reversal of the residual muscle block with glycopyrrolate and neostigmine, her recovery was adequate and satisfactory. She was shifted to a high dependency unit (HDU) for further management, where she received one more unit of whole blood along with paracetamol and nalbuphine as analgesics in the immediate postoperative period. The patient did not require any further vasopressor, inotropic, or chronotropic support. The rest of her HDU and hospital stay was uneventful for her and her baby. Repeat investigation on the postoperative fifth day found an increase in hemoglobin level (7.2 gm/dL) with RFT, LFT, and serum (Sr) electrolytes at normal levels. Baby and mother were discharged on postoperative day six.

Case 2

A 26-year-old woman of short stature, primi-gravida at term, came to our emergency department in labor on October 22, 2021. She did not previously attend any ANC anywhere else. Her general condition was poor, as she exhibited severe pallor along with slight tachycardia and borderline high blood pressure of 138/91 mmHg. Other systemic examinations were normal. Her FHS was found to be less than 80/min with a fetal Doppler; however, there were no signs suggestive of any internal or external hemorrhage. To add to the complication, she was assessed as having cephalon pelvic disproportion (CPD). Therefore, in view of the severe fetal distress and CPD, an emergency C-section was planned. As she was hemodynamically stable, she was planned for rapid-sequence spinal anesthesia, where the block procedure was performed with minimum required monitoring equipment and drugs for performing spinal anesthesia after informed consent. The block was performed quickly in a sitting position with a local anesthetic agent, 0.5% hyperbaric bupivacaine 08 mg, and a block level of T8 was confirmed after three minutes. Meanwhile, blood investigation revealed a very low Hb% (3.5 gm/dL), slightly raised liver enzymes, and normal RFT and basic coagulation profile. Her intraoperative vitals remained stable, and no vasopressor support was required. A whole blood transfusion was initiated as soon as it was made available during the surgery along with 750 mL of intravenous Ringers' lactate (RL). At C-section, a moderately depressed baby was delivered with a weight of 1.175 kg, who was immediately resuscitated and kept under neonatal intensive care unit (NICU) care at our referral center. During the immediate postoperative period in HDU and thereafter, her vitals and urine output continued to remain stable. The patient was further transfused with two units of whole blood during her HDU stay. Follow-up postoperative investigation done on the fifth day reported a better Hb% (8.2 mg/dL) level with normal RFT, LFT, and Sr electrolytes, and investigations for malaria and sickling were reported to be negative. However, her peripheral blood smear examination was suggestive of iron deficiency anemia. The baby was discharged from the NICU on the third day, and the rest of the patient’s hospital stay was uneventful.

Case 3

An 18-year-old woman, primi at term, reported to our hospital on June 10, 2023, with a history of loss of fetal movement, fever, and two to three episodes of generalized seizure over the previous two days. On examination, she was found irritable, restless, confused, and moving all four limbs. She had pallor, icterus, and edema, along with mild tachycardia, borderline high blood pressure (146/106 mmHg), and bilateral basal crepitation. Fetal cardiac activity was found to be absent with a handheld Doppler. She had hyperreflexia and bilateral plantar flexor responses. Investigation revealed altered kidney and LFTs with trace protein and numerous red blood cells in her urine (Table 1).

**Table 1:** Improvement of Hb%, kidney, and LFT parameters during postoperative HDU stay in Case 3 Hb%: hemoglobin level, Hct%: hematocrit, RBS: random blood sugar, eGfr: estimated glomerular filtration rate, ALT: alanine aminotransferase, AST: aspartate aminotransferase, HDU: high dependency unit, LFT: liver function test

Blood investigation	Day 1	Day 2	Day 3	Day 4	Day 5	Day 7	Reference range
Hb% gm/dL	12.5	11.0	6.9	5.0	6.2	6.9	11.5-16.5
Total count/cumm	12700	21500	13800	10800	12900	13900	4000-11000
Hct%	39.9	29.1	28.7	25.1	23.4	26.3	35-50
Plat count/cumm	105000	120000	108000	114000	106000	102000	150000-450000
RBS mg/dL	166	116	82	98	91	86	110-145
Urea mg/dL	45	56	118	51	64	55	18-45
Creatinine mg/dL	0.98	1.49	2.14	1.59	1.28	1.35	0.5-1.5
eGfr mL/min	86	71	34	84	81	79	90-20
Bilirubin (T) mg/dL	6.65	3.03	1.56	1.70	1.68	1.5	0.2-1.0
Bilirubin (D) mg/dL	3.40	1.68	0.68	0.89	0.86	0.76	0.1-.5
ALT u/L	368	274	245	131	61	97	5-45
AST u/L	220	117	132	83	46	47	0-35

Malaria was excluded, with this region being a malaria-endemic zone. Based on the absence of a history of any spontaneous bleeding or any pruritic patches on physical examination and a history of convulsion over the previous two days, she was considered to be a case of eclampsia. She was started on supportive treatment with antibiotics, O2 inhalation, anticonvulsants, and MgSO4 (Pritchard’s regimen). Further assessment was suggestive of a breech presentation and with good cervical effacement. Therefore, a vaginal delivery trial was considered. However, due to a contracted pelvis, only the trunk of the baby could be delivered. An urgent C-section was planned to deliver the after-coming head of the baby later in the evening on the same day. The high risk of surgery under anesthesia and the poor prognosis of the mother were explained to the responsible party accompanying the patient. At the time of pre-anesthetic assessment, the patient had tachycardia, hypotension, low oxygen saturation (84%), and basal crepitation possibly due to pulmonary edema. General anesthesia with rapid-sequence intubation was planned, and measures for aspiration prophylaxis and preoxygenation for three minutes were given. Induction was carried out with ketamine 50 mg and succinylcholine 50 mg. Cricoid pressure was applied and continued until successful intubation was confirmed with ETCO2. For maintenance, N2O+O2(1:1), relaxant atracurium 25 mg, and analgesic fentanyl 50 mcg were given. Although intraoperatively adequate fluid (500 mL RL, 500 mL colloid, and one unit of whole blood) was transfused, noradrenaline infusion support was also initiated to maintain her hemodynamics. Fluid administration was roughly guided by hemodynamic monitoring, blood loss, and urine output.

Surgery was completed within one hour, but she continued to be hemodynamically unstable and unable to maintain an oxygen saturation above 85%. Therefore, she was continued with ventilatory support under assist control mode with a tidal volume of 350 mL, a positive end-expiratory pressure of 8 cmH2O, a respiratory rate of 16/min, a fractional inspired oxygen (FiO2) of 50%, and midazolam infusion, and shifted to HDU. Paracetamol as an analgesic along with noradrenaline infusion in a titrated dose and transfusion of an additional two units of whole blood were advised. On the following postoperative day, her vitals were stable without any support, and her urine output was adequate, but her continuing hematuria was a concern. Repeat blood investigation was suggestive of a decreasing trend of Hb%, a persistently high total leucocytic count, and slightly deranged liver and kidney function tests. Her ABG was normal (Table 2). Her ventilatory support was changed to spontaneous timed weaning mode with an inspiratory positive airway pressure of 18 cmH2O, an expiratory positive airway pressure of 6 cm H2O, and a FiO2 of 30%. Subsequently, on June 13, 2023, she was extubated and was able to maintain oxygen saturation above 95% with O2 inhalation, which was discontinued later. Frusemide, tranexamic acid 500 mg till the fifth postoperative day, whole blood transfusion, and other supportive therapy were continued. During her stay, her kidney and LFTs continued to improve (Table 1). Resolution of hematuria was also observed. The deranged kidney function test was possibly due to hypoperfusion of the kidney due to decreased prehospital fluid intake and eclampsia-related glomerular endotheliosis, and hematuria was possibly due to bladder wall injury during the obstructed labor. She had received a total of five units of blood transfusion during her postoperative HDU stay. Her hemoglobin also improved to 6.9 gm/dL. She was shifted to the general ward and discharged on the ninth day of her hospital stay.

**Table 2:** Serial ABG during ventilatory support for Case 3 ABG: arterial blood gas, PCO2: partial pressure of carbon dioxide, PO2: partial pressure of oxygen, BEecf: base excess extracellular fluid, HCO3: bicarbonate, sO2: oxygen saturation, iCa: ionized calcium

ABG	Day 1	Day 2	Day 3	Day 4	Reference range
pH	7.46	7.45	7.38	7.34	7.31-7.41
PCO2 mmHg	24.3	35.1	32.9	32.1	41-51
PO2 mmHg	126	144	156	106	80-105
BEecf mmol/L	−7	−1	−3	−4	−2-3
HCO3 mmol/L	17.3	23.4	22.4	17.4	23.0-28.0
sO2%	99	99	96	100	95-98
Na mmol/L	128	137	139	137	141-146
K mmol/L	3.89	4.1	3.99	4.0	3.5-4.9
iCa mmol/L	1.04	0.9	0.9	1.03	1.12-1.32

Case 4

A 24-year-old woman, second gravida, with a previous C-section two years prior was admitted with labor pain and leaking per vagina on November 17, 2023. She had no record available with her regarding any ANC done previously during this pregnancy. Her last menstrual cycle date also could not be elicited. She was hemodynamically stable, and other systemic examinations were also normal. She was in the first stage of labor with her cervix fully effaced and dilated with a deep transverse arrest presentation. Her FHS was recorded as 138/min. Blood investigations of her kidney function, liver function, and basic coagulation profile were normal except for a low Hb%. An emergency C-section was planned, and patient preparation and immediate transfer to the operating theater were advised. However, after receiving the patient in the operation theater, she was found to have profuse perspiration, tachycardia with a pulse rate of 145/min, and an exceptionally low blood pressure of 64/42 mmHg. She was drowsy and lethargic. FHSs were not audible, and it was confirmed by a handheld Doppler machine and upon abdominal examination that fetal parts were also palpable. Resuscitation with fluid (crystalloid and colloid) and a vasopressor was started immediately in the patient's receiving area. The possibility of rupture of the uterus on her transit to the operation theater was highly suspected. High-risk and guarded prognoses were explained to the patient and her husband. The blood bank was informed of the urgent requirement for compatible whole blood. Following resuscitation, her vitals slightly improved, with a pulse rate of 158/min and a low blood pressure of 85/47 mmHg. For premedication and aspiration prophylaxis, metoclopramide, ondansetron, pantoprazole, PCM, and glycopyrrolate were given intravenously to the patient. Preoxygenation for three minutes followed by induction was done with intravenous propofol 1% 50 mg, ketamine 50 mg, and succinylcholine 75 mg. Cricoid pressure was applied and continued until intubation was confirmed with ETCO2. Maintenance of anesthesia was done with N2O/O2 with a ratio of 1:1 along with fentanyl 50 mcg and vecuronium 2 mg. Tranexamic acid was also added, and noradrenaline infusion was also started to further maintain her hemodynamics. She was further transfused with 500 mL of crystalloid and one unit of whole blood. A stillborn baby was delivered, and surgery was completed within approximately one hour. Reversal was done with glycopyrrolate and neostigmine. After the reversal, recovery was adequate. Postoperatively, her vitals were stable, and she was able to maintain oxygen saturation. Subsequently, she was shifted to HDU. Noradrenaline support was titrated and withdrawn as she continued to be hemodynamically stable thereafter. Her arterial blood gas (ABG) was normal except for low hematocrit and Hb% levels. Postoperatively, she received two units of whole blood transfusion, along with analgesics (paracetamol and pentazocine), antibiotics, and other supportive care. On postoperative day two, her RFT parameters were also found to be normal. She was shifted from HDU on day three and later discharged on postoperative day seven.

Case 5

A 30-year-old woman from a nearby village with a previous history of two lower uterine c-sections, three and seven years ago, was admitted on March 9, 2024, with a history of amenorrhea for the last three months and vaginal bleeding for the last 15 days. On admission, her vitals were stable. The investigation was suggestive of low Hb% and hematocrit levels. Her coagulation profile, liver function, kidney function, and electrolytes were normal. Initially, because of her vaginal bleeding and low Hb%, a differential diagnosis of spontaneous abortion was made, and one unit of whole blood was advised. Ultrasound of the lower abdomen was suggestive of a bulky, empty uterus with a well-defined gestational sac in the pouch of Douglas (POD). The fetus had a crown-rump length of 12 mm and visible fetal heart activity.

**Figure 1:**
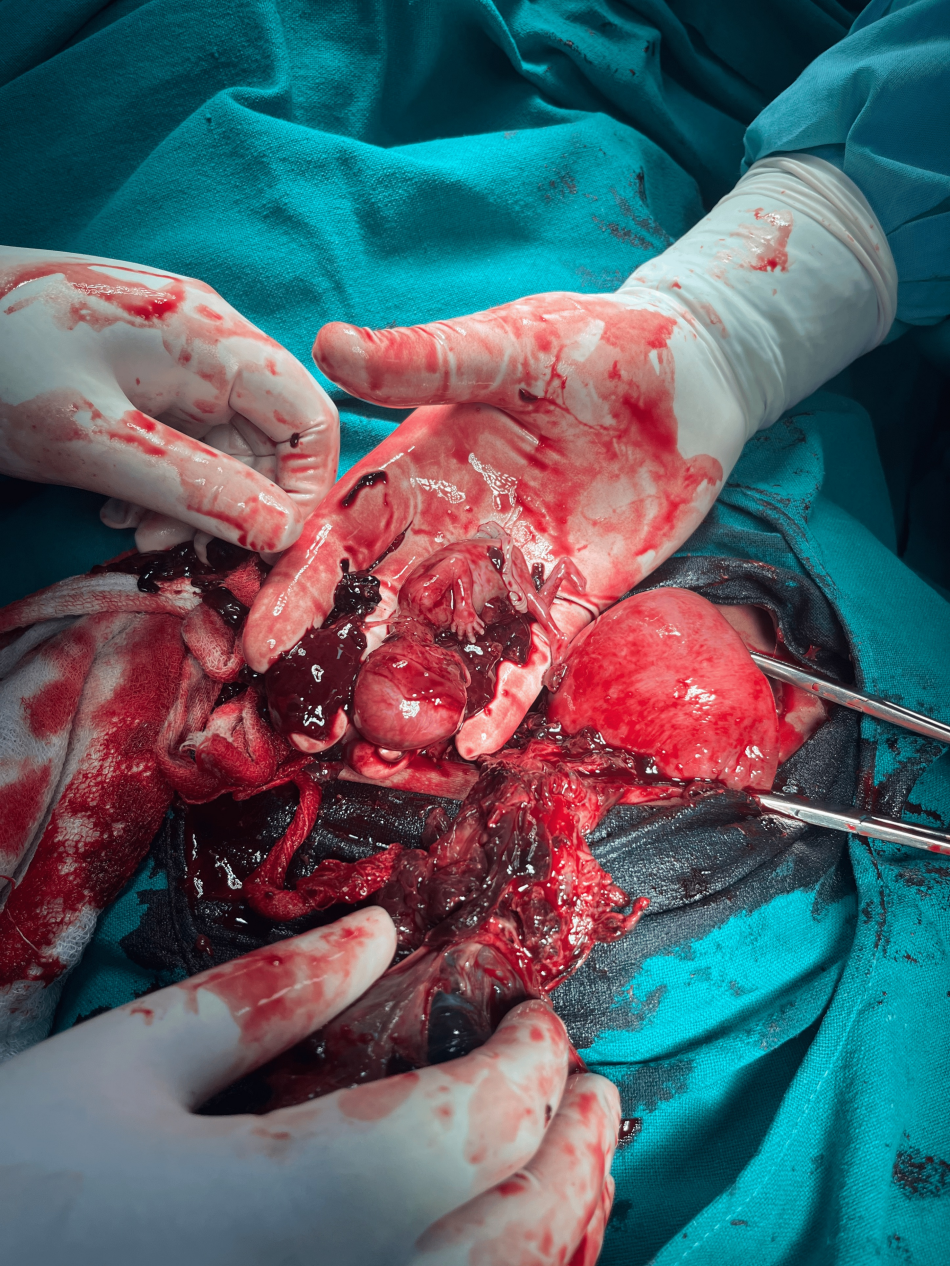
Intraoperative extrauterine fetus with placenta

The patient was posted for urgent laparotomy for removal of the extra uterine mass. However, during pre-anesthetic assessment, the patient was found to have a fever, but her vitals were normal. Abdominal examination was suggestive of definite localized tenderness over the lower quadrant. However, other systemic examinations were normal. She had received pantoprazole, metoclopramide, and ondansetron for aspiration prophylaxis. Two wide-bore 16G intravenous cannulations were secured. For general anesthesia, premedication with midazolam, fentanyl, paracetamol, and tranexamic acid (1 g) was given intravenously. An 18-G epidural catheter was placed in the epidural space at the L3-L4 vertebrae level using a 1 6G Tuohy needle under aseptic and antiseptic measures. Pre-induction, her vitals were stable except for tachycardia. After preoxygenation for three minutes, induction was done with propofol 1% and succinylcholine. Following induction, intubation was carried out with a 7-mm endotracheal tube, and placement was confirmed with ETCO2. For maintenance of anesthesia, N2O+O2 (1:1 ratio) and sevoflurane and vecuronium as muscle relaxants were used. At laparotomy, an already ruptured sac with the placenta densely adhered to broad ligaments and POD was found along with a fetus 10-12 cm in size, which was removed (Figure 1). During the removal, there was significant blood loss, and two units of whole blood were transfused.

Recovery was adequate after reversal with a combination of glycopyrrolate and neostigmine. Post-recovery, her vitals were stable, and her urine output was adequate. She was shifted to HDU for postoperative care and management. The pain was managed with a combined infusion of 0.125% bupivacaine and fentanyl delivered by the epidural catheter placed in situ, which was later removed on the third postoperative day. Laboratory blood investigation showed an increased Hb% of 9.9 gm/dL and normal kidney and LFTs. Subsequently, she shifted from HDU and was discharged on the seventh postoperative day.

## Discussion

Management of obstetrical emergencies requires a multidisciplinary approach. The guidelines for resuscitation (airway, breathing, and circulation) of obstetric emergencies are the same as those of any other emergency, with the distinction of two lives being jeopardized. The goals of these resuscitations are a treatment of shock with whole blood transfusion and vasopressors to maintain hemodynamic stability while minimizing the risk of hypoxemia for both mother and fetus.

For emergency C-sections, where there is a threat to the mother or baby, general anesthesia is the best option. Acid-aspiration prophylaxis with agents such as proton pump inhibitors, metoclopramide, ondansetron, and sodium citrate, along with cricoid pressure and oxygen flow at a Pmax of 20 cm H2O (10 N increasing to 30-40 N after induction until intubation is confirmed with ETCO2) are to be routinely used. Minimum monitoring devices for monitoring oxygenation, ventilation, and circulation are to be ensured [6], and gas monitoring for inhalational agents is also preferable. There is a significant increase in oxygen demand during pregnancy due to a 15% increase in metabolic rate and a 20% increase in oxygen consumption. Furthermore, diaphragmatic elevation in late pregnancy also results in a decrease in functional reserve capacity. These changes lead to a reduced oxygen reserve, a decrease in apnea time without desaturation, and thus an increased risk of hypoxemia. During induction, the patient was positioned at a left lateral tilt (for fetal wellness and resuscitation) with the head propped up 30-45 degrees to slightly increase the functional reserve capacity and reduce reflux. Preoxygenation for three minutes or four to five full-tidal-volume breaths prior to induction of anesthesia will increase the oxygen reserve of the mother and prevent hypoxia during the process of intubation.

Intubation in parturients has a high rate of failure owing to increased body weight, edema of the airway, and increased size of the tongue and breasts. The Difficult Airway Association recommends a maximum of three attempts of intubating the patient, each with a change of technique and blade used; they also recommend the use of a video laryngoscope after the first failed attempt, and the third attempt should be carried out by the senior-most anesthesiologist. Supraglottic airway devices such as LMA ProSeal or i-GEL should be kept ready in case of cannot intubate and cannot oxygenate situations [7]. If oxygenation is maintained, surgery may be allowed to continue. In extreme circumstances, emergency front-of-neck airway access, cricothyroidotomy, or emergency tracheostomy may be required to maintain oxygenation.

The induction drug of choice for rapid sequence intubation would be a short-acting agent with minimum adverse hemodynamic effects and provide reliable anesthesia and amnesia. Thiopentone is still the agent of choice; however, for hemodynamically compromised patients, drugs like etomidate and ketamine can also be used. The direct effect of ketamine on the heart is negatively inotropic, especially in heart failure patients. However, with an intact autonomic nervous system, ketamine acts as a sympathomimetic to increase heart rate, arterial pressure, and cardiac output. The combination of sympathomimetic effects, preservation of baroreflex response, and absence of idiosyncratic adverse effects (especially impaired steroidogenesis, such as occurs with etomidate) confer distinct advantages on ketamine when used for rapid sequence intubation in hemodynamically compromised patients. Anesthesia may be preferably maintained with volatile agents like isoflurane or sevoflurane along with N2O and intermediate-acting muscle relaxants such as atracurium or vecuronium. The minimum alveolar concentration of volatile agents may be lowered to as low as 0.4-1% and adjusted to avoid hemodynamic instability and a decrease in uterine contraction.

In cases of eclampsia, the main goals are to control seizures and manage blood pressure. The presence of altered sensorium and localizing signs could be an indication of intracranial pathology and poor outcome. Regional anesthesia can be safely used if the patient with eclampsia is conscious and seizure-free. As per the American Society of Anesthesiologists, regional anesthesia may be considered safe in patients with low-dose aspirin and a platelet count of more than 75,000 per microliter. Small doses of phenylephrine (50 mg) may be used to treat hypotension with judiciously used additional IV fluid. General anesthesia is the choice for otherwise unconscious, obtunded, or patients with features suggestive of intracranial pathology. Airway edema, difficult airway management, exaggerated hypertensive responses to intubation, pulmonary edema, and interaction between magnesium sulfate and muscle relaxants are important issues. Coagulation profiling should also be advised regardless of platelet count because, during pregnancy, there is an increase in procoagulant activity by elevation in factor VII, factor VIII, factor X, and fibrinogen and a decrease in reduction in fibrinolysis and protein-s activity resulting in a hypercoagulable state. Prothrombin time or activated partial thromboplastin time is used in evaluating the coagulation status during pregnancy; however, thrombo-elastography and rotational thrombo-elastometry, used for assessing the real-time function of the platelet function, coagulation factors, fibrinogen, and fibrinolysis, are more accurate. Specific blood products or whole blood, as required and available, should also be ensured [8]. A urine output of >0.5 mL/kg can be used to roughly assess kidney perfusion. Intraoperative analgesics with paracetamol and fentanyl may be used as permissible. The timing of reversal and extubation is critical in these morbid patients, which are preferably done after the patient has recovered from the residual block and is able to generate adequate tidal volume. Ventilatory support may be required depending on the patient's condition. Postoperative care should include pain management, fluid management, and adequate component or whole blood transfusion as required. Monitoring of vitals, kidney function, liver function, and ABG with electrolytes is to be done regularly for early intervention if required.

If the mother is hemodynamically stable and conscious, a rapid sequence spinal anesthesia (RSS) procedure can be performed quickly by an experienced anesthesiologist to shorten the DDI for emergency and urgent C-sections [9]. RSS is slightly different from normal spinal anesthesia in that it is carried out with basic requirements with a single attempt and allows for surgery as soon as the block is achieved up to the T10 level, thus avoiding the risk associated with general anesthesia for mother and baby [10]. The drug of choice for rapid-sequence spinal anesthesia is a maximum of 2.5 ml of 0.5% hyperbaric bupivacaine with added small doses of fentanyl (maximum 25 mcg), which may increase the onset and duration of the block and decrease the requirement of a local anesthetic agent, providing more hemodynamic stability to the patient [11]. RSS has its own limitations and is to be avoided and contraindicated in parturients with obesity, spinal deformity, coagulopathies of pregnancy, infection at the site of injection, and altered sensorium with or without localizing signs. The neonatologist must be informed about the addition of any opioids. However, the care team should be ready for conversion to general anesthesia if there is an inadequate block or even a slight delay in its onset. Epidural top-up in parturients with a previously well-placed epidural catheter for labor analgesia may also be used but is limited by its delayed onset to the depths required for surgical anesthesia.

In cases of maternal obstetric hemorrhage (i.e., blood loss from the uterus or genital tract >1500 mL; decrease in hemoglobin of >4 g/dL; or acute blood loss requiring transfusion of more than four units of blood), management goals are to restore the blood volume, maintain hemodynamics, monitor, correct the coagulation defect, if any, and evaluate the response to treatment [12].

Antibiotic prophylaxis as per antibiotic policy, standard operating procedure for infection control, operation theater sterilization, surgical safety checklist, and regular training of the staff at all levels in universal infection prevention practices decrease surgical site and other postpartum maternal infection rates.

For all cases presented here, a quick pre-anesthetic assessment regarding hemodynamic stability, airway, and other systemic disease was done, and minimum investigation orders were advised, including complete blood count, blood grouping and crossmatching, RFT, LFT, bleeding time, and clotting time. Time did not permit us to carry out other radiological and coagulation profile investigations, as they were to be sent to our higher-level center. The resuscitation of both mother and baby was started once the quick pre-anesthetic assessment was completed. Minimum blood order scheduling, along with the urgency of the requirement, was clearly communicated to the blood bank. Other measures for the treatment of shock, a difficult airway plan, and securing wide-bore IV access with a second line were also ensured. In utero fetal resuscitation with patient positioning (left lateral), oxygen, and tocolysis were also ensured. All patients posted for C-sections were prepared for general anesthesia irrespective of indication, as all parturients are at considerable risk of aspiration. Table 3 shows the comparison of anesthetic management outcomes of the five C-section cases.

**Table 3:** Comparison of anesthetic management outcomes of the five C-section cases ANC: antenatal checkup, C-section: cesarean section, IUD: intrauterine death, C-section: cesarean section

Case	ANC	Indication	Anesthesia	Outcome
1	None	Second gravida with retroplacental hematoma (emergency)	General anesthesia	Mother and baby survived
2	None	Primi with fetal distress and severe anemia (emergency)	Rapid-sequence spinal anesthesia	Mother and baby survived
3	None	Primi eclampsia with contracted pelvis for delivery of the after-coming head of IUD fetus (urgent)	General anesthesia	Mother survived
4	None	Post C-section, ruptured uterus, IUD (emergency)	General anesthesia	Mother survived
5	None	Twice post C-Section with ruptured extrauterine at 12 weeks of pregnancy	General anesthesia	Mother survived

## Conclusions

The anesthetic management of emergency obstetric cases, often presenting late with a significant threat to the life of the mother or fetus, remains a formidable challenge. The primary goal of ensuring hemodynamic stability and adequate tissue oxygenation and mitigating the impact of complications can reduce the mortality and morbidity associated with these cases. Although the patients discussed in this case series were from nearby villages, the broader population in this remote mining area is reluctant to attend health care facilities available to them due to illiteracy, lack of awareness, and poverty. To improve overall outcomes for both mothers and fetuses, it is also crucial for us to engage with this population more proactively, promoting regular ANCs to optimize pregnancy-related care and create awareness regarding institutional delivery.
